# Methylenetetrahydrofolate Reductase C677T Gene Variant in Relation to Body Mass Index and Folate Concentration in a Polish Population

**DOI:** 10.3390/biomedicines10123140

**Published:** 2022-12-06

**Authors:** Małgorzata Wrzosek, Klaudia Ślusarczyk

**Affiliations:** 1 Department of Biochemistry and Pharmacogenomics, Medical University of Warsaw, 1 Banacha St., 02-097 Warsaw, Poland; 2 Centre for Preclinical Research, Medical University of Warsaw, 1B Banacha St., 02-097 Warsaw, Poland; 3 Department of Medical Genetics, Institute of Mother and Child, 17a Kasprzaka St., 01-211 Warsaw, Poland

**Keywords:** BMI, methylenetetrahydrofolate reductase, folate, *MTHFR* C677T

## Abstract

Background: Methylenetetrahydrofolate reductase (MTHFR) is an enzyme responsible for producing an active form of folate. The *MTHFR* T677T genotype may have adverse health effects associated with weight gain and a reduction in folate availability. Aim: In this study, we examined the influence of the *MTHFR* C677T variant on BMI and determined its role as a risk factor for obesity. We also assessed the relationships between the *MTHFR* C677T genotype and folate and vitamin B_12_ concentrations in subjects before and after radical weight loss. Methods: The study group consisted of 1712 Caucasian adults of Polish nationality with a wide range of body mass indexes (BMIs). DNA was extracted from the blood, and the C677T variant was tested with RFLP-PCR and real-time-PCR. Results: There was no significant influence found for the *MTHFR* 677 TT genotype on BMI or the obesity risk in codominant, dominant or recessive inheritance models. Individuals with obesity and the TT genotype had significantly lower folate concentrations. After radical weight reduction, the impact of the risk genotype, as well as homeostasis between folate and vitamin B_12_ in TT homozygotes, seems to be attenuated. Conclusion: Although the *MTHFR* 677TT genotype is not directly related to a higher BMI in Polish adults, it has a detrimental effect on folate concentrations in individuals with high BMI values.

## 1. Introduction

Methylenetetrahydrofolate reductase (MTHFR) plays a pivotal role in folate, homocysteine (Hcy) and methionine metabolism. This enzyme catalyzes the conversion of 5,10-methylenetetrahydrofolate into 5-methyltetrahydrofolate (5-MTHF, 5-methyl-THF), which is an active folate form and a methyl donor for DNA methylation or for the conversion of Hcy into methionine. An *MTHFR* polymorphism leads to enzyme dysfunction, possibly resulting in higher Hcy levels and disturbances in one-carbon metabolism. Thus, *MTHFR* gene variants have been linked to many metabolic pathologies, including gestational weight gain [1]. The *MTHFR* gene has 12 exons and is located on chromosome 1 (1p36.22). The promoter region of this gene does not have a TATA box [2,3]. The MTHFR enzyme is composed of catalytic and regulatory domains and is inhibited by binding to S-adenozylomethionine (SAM), dihydrofolate (DHF) and its polyglutamate analogs. MTHFR is also regulated by multisite phosphorylation in its serine-rich region, which decreases enzyme activity [2,3,4].

There are numerous genetic variants that can affect folate metabolism. The most investigated variant of the *MTHFR* gene is the C677T polymorphism (rs1801133). In 1988, Kang et al. showed the decreased activity and higher thermolability of the MTHFR enzyme in the lymphocytes of patients with ischemic heart disease [5]. Patients with up to a 75% decrease in the activity of the enzyme had increased concentrations of total Hcy [6]. Subsequently, Frosst et al. (1995) noted that cytosine (C) is replaced by thymine (T), which leads to the substitution of alanine with valine at position 222 in the catalytic domain of methylenetetrahydrofolate reductase. This change influences how it binds to flavin adenine dinucleotide (FAD), which acts as a cofactor of the enzyme [7]. It was shown that, compared to those with the 677CC genotype, *MTHFR* 677TT homozygotes have only about 30% of the normal enzyme activity, while heterozygotes (CT) have about 65–70% [7,8].

**Scheme 1:**
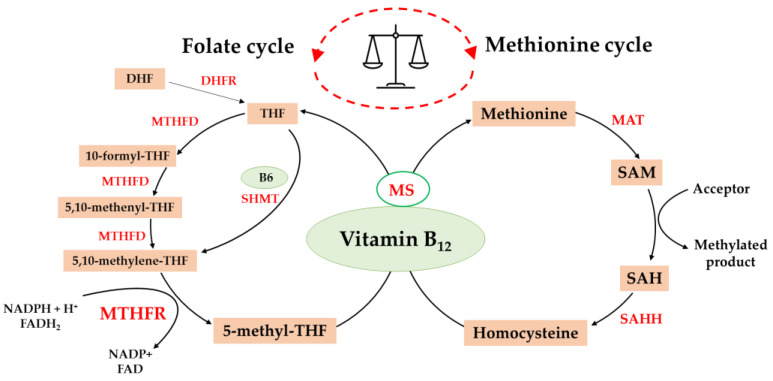
The balance between the folate cycle and methionine cycle is affected by vitamin B_12_. DHF, dihydrofolate; THF, tetrahydrofolate; MTHFD, methylenetetrahydrofolate dehydrogenase; SHMT, serine hydroxymethyltransferase; DHFR, dihydrofolate reductase; MTHFR, methylenetetrahydrofolate reductase; MS, methionine synthase; MAT, methionine adenosyltransferase; SAM, S-adenosylmethionine; SAH, S-adenosylhomocysteine; SAHH, S-adenosylhomocysteine hydrolase; NADPH, nicotinamide adenine dinucleotide phosphate; FADH_2_, dihydroflavine-adenine dinucleotide.

Folates are biologically active, water-soluble compounds found in leafy greens and as synthetic folic acid. They are very important cofactors, taking part in many essential biochemical processes. Chemically, the structure of folates is based on a pterin (2-amino-4-hydroxy-pteridine) heterocyclic ring, a p-aminobenzoyl group and a glutamate residue [9,10]. There are about 40 natural folate compounds. They differ from each other in the degree of oxidation of mono-carbon groups, such as methyl (-CH_3_), methylene (-CH_2_-), methenyl (-CH=), formyl (-CH=O) and formimino (-CH=NH), as well as in the number of glutamic acid residues attached [11]. Folates supplied by the diet are metabolized to 5-MTHF in several steps. 5-MTHF is the most active derivative and has the highest concentration in plasma. Due to its participation in one-carbon metabolism, a complex interrelationship occurs between folate and vitamin B_12_ (Scheme 1). Briefly, folates occurring in food and in supplemented folic acid are absorbed through the mucosa in the small intestine by a transporter that carries both oxidized and reduced folates as monoglutamates [12]; i.e., before their absorption, polyglutamate forms are hydrolyzed by specific enzymes. Conversion to polyglutamate is required for folate to be biologically active. In the cytosol, the first reaction is the reduction to dihydrofolate (DHF) and subsequently to tetrahydrofolate (THF) [13]. The conversion of 10-formyl-THF and 5,10-methenyl-THF is catalyzed by methylenetetrahydrofolate dehydrogenase (MTHFD). THF can also be directly converted into 5,10-methylene-THF by SHMT (serine hydroxymethyltransferase) through the vitamin-B_6_-dependent pathway. Then, 5,10-methylene-THF is converted into 5-MTHF by MTHFR. The irreversible reaction catalyzed by the MTHFR enzyme has a crucial role in folate metabolism, as it regulates the bioavailability of 5-MTHF, which is necessary for methionine synthesis. Methionine is transformed into SAM, which is the main methyl donor for a variety of processes. Vitamin B_12_ is converted to methylcobalamin, which serves as a cofactor for methionine synthase (MS). Both 5-MTHF and methylcobalamin are crucial for the synthesis of methionine from Hcy. The methyl group is donated by 5-MTHF. If the vitamin B_12_ concentration is too low, MS is inactivated, and functional folate deficiency occurs. This is because folates become increasingly “stuck” as 5-MTHF and cannot be utilized for regeneration [14,15].

Disturbances in Hcy metabolism have more often been recognized in people with obesity [16,17]. Previous studies have assessed the relationship between the *MTHFR* C677T polymorphism and metabolic syndrome. Most of them have shown a significant positive association in people in China [18], but evidence from other populations is conflicting [19]. Thus, in the present study, we examined the influence of the *MTHFR* C677T variant on BMI and determined its role as an obesity risk factor in a group consisting of 1712 individuals from a *Caucasian* population. The C677T variant results in lower MTHFR enzyme activity, which leads to a low serum folate status. Clinical evidence indicated that serum folate levels were significantly lower among those with the 677 TT genotype than among C allele carriers [20]. However, there is a lack of data describing any potential associations between radical weight loss, folate status, the balance of folate and vitamin B_12_, and the methylenetetrahydrofolate reductase gene polymorphism. Thus, the second aim of this study was to assess any relationships between the *MTHFR* C677T variant and the serum folate and vitamin B_12_ levels in a group of individuals before and after radical weight loss.

## 2. Materials and Methods

### 2.1. Study Population

For this study, 1712 unrelated adults of Polish nationality, with a mean age of 44.8 ± 12.6 years, were recruited between September 2012 and September 2018, as reported elsewhere [21,22]. The study population consisted of 518 non-obese individuals (healthy controls) and 1194 patients with a BMI above 30 kg/m^2^. The patients and the controls were frequency-matched according to age and gender. A group of participants with class II/III obesity was assigned to bariatric surgery, as reported elsewhere [22], and follow-up examinations were performed one year after the operation in 354 of these participants. The study was approved by the Institutional Bioethics Committees (KB/127/2012 and KB/67/2017 at the Medical University of Warsaw, Warsaw, Poland). Written informed consent was obtained from each participant after a full explanation of the study. The study was carried out in accordance with the principles of the Declaration of Helsinki. A detailed clinical history was obtained and a full physical examination was performed for each patient. Anthropometric measurements were taken, and body mass index (BMI) was calculated for all subjects. Obesity was diagnosed when BMI ≥ 30 kg/m^2^. Participants were classified as hypertensive if their average blood pressure measured ≥140/90 mm Hg or if they were on hypertensive medication at the time of the interview due to a previous diagnosis of hypertension [21]. A review of medical records was performed, and if a physician had diagnosed diabetes, glucose-lowering medications had been prescribed, and diabetes was confirmed by current medical examination, the patient was classified as diabetic [23]. If participants had been diagnosed according to the National Cholesterol Education Program-Adult Treatment Panel III (ATP III) guidelines [24] and/or reported using lipid-lowering medication, they were classified as having dyslipidemia.

### 2.2. Measurements of Biomarkers

Overnight peripheral fasting blood samples were taken from all subjects. The sera were isolated, used for analyses or stored at −80 °C. All samples were analyzed by specialist clinical laboratory personnel. The biochemical analyses included measurements of glucose, HbA1c (%), folate, vitamin B_12_, total cholesterol, high-density lipoprotein cholesterol (HDL-C), low-density lipoprotein cholesterol (LDL-C), triglycerides, creatinine (CRE), C-reactive protein (CRP), 25(OH)D, aspartate aminotransferase (ASP), alanine aminotransferase (ALT) and erythrocyte sedimentation rate (ESR). Serum levels of interleukin 6 (IL-6) were determined with an enzyme-linked immunosorbent assay (ELISA), using the Diaclone Human IL-6 High Sensitivity ELISA kit (Diaclone SAS, Besancon Cedex, France). The laboratory staff was kept unaware of the genotypes of the subjects.

### 2.3. Genotyping of the MTHFR C677T Polymorphism

**Figure 1:**
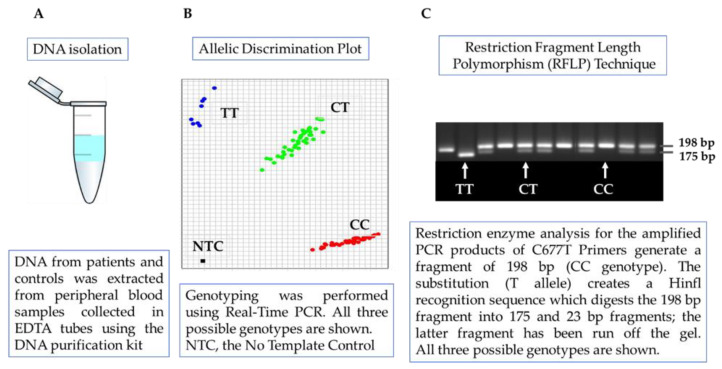
The design of the experiment: (**A**) DNA isolation. (**B**) The Real-Time PCR method. (**C**) Polymerase chain reaction (PCR) followed by restriction digestion analysis (own measurements).

Genomic DNA from patients and controls was extracted and purified from peripheral blood samples collected in EDTA tubes using the Blood Mini genomic DNA kit (A&A Biotechnology, Gdynia, Poland) according to the manufacturer’s instructions and quantified by a UV-Vis spectrophotometer (Quawell Q3000, Quawell Technology Inc., San Jose, CA, USA). Analysis of the *MTHFR* C677T polymorphism was performed by polymerase chain reaction (PCR) followed by restriction digestion analysis. Amplification was performed using the forward primer, 5′-TGA AGG AGA AGG TGT CTG CGG GA-3′, and the reverse primer, 5′-AGG ACG GTG CGG TGA GAG TG-3′, which generate a fragment of 198 bp. Each PCR reaction of 25 μL involves 1U BIOTAQ™ polymerase (Bioline, Luckenwalde, Germany), 1× PCR buffer (10× NH_4_ Reaction Buffer), 1.5 mM magnesium chloride solution (50 mM MgCl_2_), 400 µM dNTP (Thermo Scientific™, Waltham, MA, USA) and 0.4 pmol of each primer. Samples were amplified using the following PCR conditions: 5 min of initial denaturation at 94 °C, followed by 35 cycles of 94 °C for 30 s, 60 °C for 30 s, and 72 °C for 30 s, with a final extension at 72 °C for 3 min. The amplified PCR product was digested with HinfI restriction enzymes (Thermo Scientific™, Waltham, MA, USA). The digestion reactions contained 20 μL of PCR product, 2.5 μL of R buffer (supplied with enzyme), 0.5 μL of restriction enzyme, and 2 μL of nuclease-free water. Restriction analysis was performed for 4 h at 37 °C. The digestion products were analyzed by electrophoresis on ethidium bromide-stained 2.5% agarose gel and visualized under UV light (ChemiDoc MP, Bio-Rad, Hercules, CA, USA). The digestion fragment sizes were a single 198 bp band for the 677 CC genotype; 198 bp, 175 bp and 23 bp for 677 CT; and 175 bp and 23 bp for 677 TT. The genotypes were tested twice, and genotyping was 100% concordant. A second genotyping was performed using a pre-validated TaqMan Assay designed by Life Technologies (Assay ID:C___1202883_20; SNP ID:rs1801133). The experimental schemes are shown in Figure 1. Probes were labeled with VIC and FAM fluorochromes to identify the homozygotes and heterozygotes. Reactions were conducted in 96-well plates with a total volume of 12 μL. We used 2 ng of genomic DNA, TaqMan Genotyping Master Mix 1× (Life Technologies) and TaqMan Genotyping Assay 1×. PCR cycling conditions were 95 °C for 10 min, 40 cycles of 95 °C for 15 s, and 60 °C for 1 min. After amplification, the fluorescence data were captured using a ViiA7 Real-Time PCR System (Applied Biosystems, Life Technologies, Waltham, MA, USA).

### 2.4. Statistical Analyses

Categorical variables are reported as frequencies and percentages. The parameters measured as continuous variables were compared by ANOVA, Student’s, Kruskal–Wallis or Mann–Whitney test according to the distribution. Continuous variables are presented as means with standard deviations to allow better comparison with other publications. Categorical variables were compared using the Chi-square test. The distributions of *MTHFR* C677T genotypes were analyzed for deviations from Hardy–Weinberg equilibrium using Chi-square analysis. Unconditional logistic regression analysis was used to evaluate the associations of the studied *MTHFR* C677T variant with obesity risk. Odds ratios (ORs) and 95% confidence intervals (CIs) were calculated in both univariable and multivariable codominant, dominant and recessive models. The ability to recognize the lower folate status by the *MTHFR* 677 TT genotype was assessed by receiver operating characteristic (ROC) curves and the associated area under the curve (AUC). The optimal cut-off point was calculated as the value whose sensitivity and specificity were the greatest. A linear association between serum folate and B_12_ levels was investigated using Spearman’s rank correlation coefficient.

## 3. Results

A total of 1712 individuals (1164 women and 548 men) with a mean age of 44.8 ± 12.6 years and an average BMI of 34.1 ± 10.1 kg/m^2^ were enrolled in the study. The study population consisted of 518 non-obese individuals (healthy controls) and 1194 patients with obesity. As expected, the subjects with obesity manifested significantly higher body mass indexes, fasting glucose, total cholesterol, low-density lipoprotein cholesterol (LDL-cholesterol), triglycerides (TG), systolic blood pressure and diastolic blood pressure (DBP) but lower high-density lipoprotein cholesterol (HDL-cholesterol) (Table 1).

A BMI < 30 was found in 518 subjects, 257 were categorized as having class I obesity (BMI: 30.0–34.9 kg/m^2^), 298 had a BMI in the range of 35–39.9, and 640 had a BMI above 40 kg/m^2^. The results of the genotype distribution in the populations of these four groups (BMI < 30, obesity classes I, II and III) are shown in Table 2. The *MTHFR* 677 TT genotype was not associated with higher BMI levels across the four strata of BMI (Table 2). The distribution of the *MTHFR* C677T genotypes across the subject groups did not deviate from the Hardy-Weinberg equilibrium (*p* > 0.05).

The minor allele frequency (MAF) for the 677 T variant in the whole group was 0.31, where the genotype frequencies of CC, CT and TT accounted for 47.4%, 44.0% and 8.6%, respectively, and these are consistent with those previously reported for Polish [25], UK [26] and Bosnia and Herzegovina populations [27]. Next, we examined the genetic associations between the *MTHFR* C677T variant and obesity risk in both univariable and multivariable dominant, recessive and codominant models. A BMI above 30 kg/m^2^ was not associated with the *MTHFR* C677T polymorphism in the univariable model, nor was it associated after adjustments for hypertension, diabetes and dyslipidemia (Table 3).

As the *MTHFR* C677T polymorphism affects folate status, a relation between serum folate concentrations and the studied variant was also examined in 908 of the patients with high BMI values and excess adiposity, who were assigned to bariatric surgery and underwent serum B_12_ and folate concentration testing (Table 4). Based on their *MTHFR* C677T genotype, we divided patients into three groups, TT vs. CT vs. CC, and the characteristics of the subjects are presented in Table 4. The individuals carrying the *MTHFR* 677 TT genotype had significantly lower concentrations of serum folate (6.6 ± 2.9 ng/mL) compared to the subjects with the CT genotype (8.0 ± 3.7 ng/mL) and the CC genotype (8.2 ± 3.7 ng/mL) (Table 4). Receiver operating characteristic (ROC) curves were used to evaluate the 677 TT genotype as a marker of lower folate levels in individuals with high BMI values (class II/III obesity). Folate concentrations <6.72 ng/mL (according to the Youden index), with a sensitivity of 67% and a specificity of 61%, were found to significantly discriminate between patients with the TT and CC genotypes. The AUC for ROC was 0.64 (95% CI 0.57–0.71, *p* = 0.0001).

Follow-up examinations were performed one year after bariatric surgery for 354 of the participants (279 women and 75 men). Individuals who missed the follow-up visit were excluded from the final analyses. Changes in anthropometric parameters and folate and B_12_ levels following radical weight loss according to the *MTHFR* C677T genotype are shown in Table 5. In the *MTHFR* 677 TT genotype group, it was found that patients’ body weight (BW) had decreased by −36.5 ± 14.4 kg, and their BMI had decreased by −12.9 ± 4.4 (kg/m^2^); similar changes were found in C allele carriers (Table 5). The mean percentage of weight loss (WL %) and the percentage of excess weight loss (ExWL %) for the whole group were 31.0 ± 8.1% and 74.3 ± 23.3%, respectively, and we did not find an influence of the *MTHFR* C677T genotype on weight reduction (Table 5). Changes in folate levels (Δ folate) and B_12_ levels (Δ B_12_) after weight loss were calculated by subtracting final concentrations from baseline values. The levels of B_12_ in subjects carrying the TT genotype decreased by −52.7 ± 165.6 (pg/mL), but the amount of change varied insubstantially among *MTHFR* C677T genotypes. However, after radical weight loss, the increase in mean serum folate levels was the highest in individuals carrying the TT genotype (Table 5).

**Figure 2:**
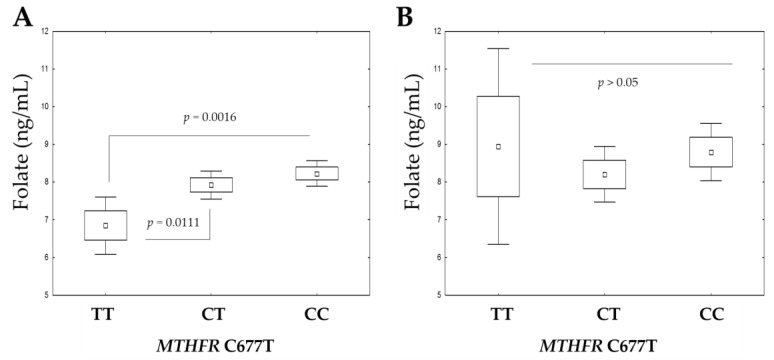
The serum folate concentrations at baseline (**A**) and after body-weight reduction (**B**) according to *MTHFR* C677T genotype. The boxplots show the mean, SE (standard error) and 1.96 × SE. ANOVA with Tukey’s multiple comparisons test was performed.

Most interestingly, the effect of the *MTHFR* C677T genotype on serum folate levels was dependent on weight. Individuals with obesity with the *MTHFR* 677 TT genotype had the lowest serum folate concentrations (Table 4). After significant weight reduction, the impact of the risk genotype seems to be attenuated (Figure 2).

**Figure 3:**
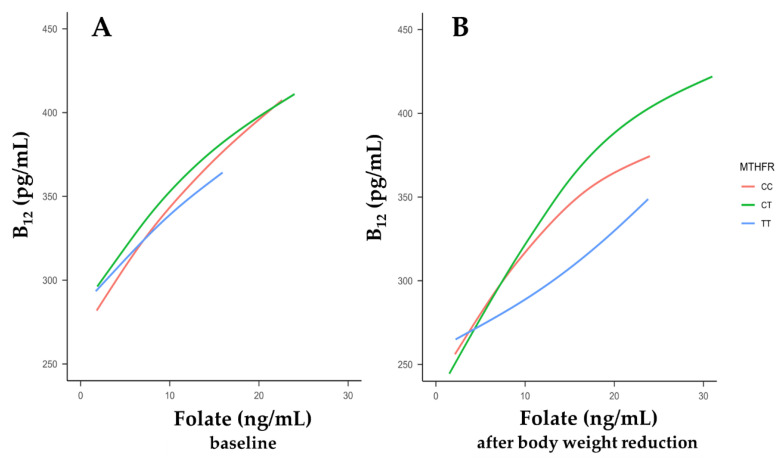
The relationships between serum vitamin B_12_ concentrations and folate levels according to the methylenetetrahydrofolate reductase (MTHFR) C677T genotype. Smoothing curves were drawn between B_12_ levels and folate levels at baseline (**A**) and after body-weight reduction (**B**).

Both folates and vitamin B_12_ have a crucial role in the maintenance of one-carbon metabolism and are responsible for many of the reactions and processes in the human body. Vitamin B_12_ enables proper and effective folate utilization. A statistically significant positive correlation between the folate and vitamin B_12_ concentrations was seen in patients with the *MTHFR* CC and CT genotypes (*p* < 0.05). This was true for both the baseline visit (Figure 3A) and the visit after body-weight reduction (Figure 3B) and can be explained by the mutual participation of folate and vitamin B_12_ in metabolic pathways.

In patients with the *MTHFR* 677 TT genotype, Spearman’s correlations did not reach significance (*p* > 0.05, Figure 3B) after body-weight reduction. Thus, in individuals with the risk-associated TT genotype, the homeostasis between serum folate and B_12_ levels may be disturbed in comparison to those with the other *MTHFR* 677 genotypes.

## 4. Discussion

About 10% of Caucasians have the *MTHFR* 677 TT genotype, which is responsible for a less-active thermolabile variant of 5, 10-methylenetetrahydrofolate reductase [7] and associated with lower folate levels [28]. In this study, patients with a BMI above 35 kg/m^2^ and with the risk-associated *MTHFR* 677 TT genotype had significantly lower concentrations of serum folate than C allele carriers. Our results are in line with the study by Bueno et al. [29], where folate serum levels were lower in 781 adults from a Spanish population with the *MTHFR* 677 TT genotype compared to the wild-type genotype. However, it cannot be excluded that the risk of low-folate status associated with the *MTHFR* 677 TT genotype may depend on a combination with other polymorphisms, as previously reported [29,30]. Folate consumption also influences folate status. Previously, serum folate concentrations were measured in young Japanese women. The intake of dietary folates was calculated based on dietary records. Even if taking in the same amounts of folates, patients with the TT genotype had lower folate concentrations. The authors suggested that people with the TT genotype consume more folates in their diet [31]. On this basis, it can be concluded that the *MTHFR* C677T variant contributes to disturbances in the folate cycle, as folate cannot be sufficiently converted into a biologically active form. Additionally, although the *MTHFR* 677 TT genotype was not directly related to BMI among the studied Polish adults, it does have a detrimental effect on folate concentrations in individuals with high BMI values. An important strength of our study is that it is the first to consider the influence of radical weight reduction on folate status according to the *MTHFR* genotype. Our data showed that the folate-lowering effect of the TT genotype was pronounced among participants with excess body weight and low baseline folate. Radical weight loss significantly modified this interaction. The mechanisms linking fat accumulation and folate deficiency remain unknown. It has previously been reported that the folate serum concentration was lower in overweight/obese people and that it was independent of the total folate intake [32,33]. Similarly, in an earlier Polish study, participants with high BMI values (n = 213) had 8.5% lower serum folate concentrations than the controls [25]. An analysis carried out by Kim et al. is in line with the findings mentioned above. The authors found a significant negative correlation between serum folate concentrations and BMI values in a group of 1462 pregnant Korean women [33]. The folate deficiency and altered folate metabolism in individuals with high body fat can be explained by the fact that adiposity is associated with systemic oxidative stress. Fat mass expansion impairs the synthesis of adipocyte-derived mediators and causes inflammation, which is commonly associated with increased ROS generation [34,35], resulting in an increased need for folates. The beneficial effect of folate is partially attributed to its maintenance of the cellular redox status by decreasing oxidative stress [36]. In our study, positive relationships were noted between levels of folate and vitamin B_12_ in patients with *MTHFR* 677 CC and CT genotypes. The metabolism of folate is associated with the action of vitamins B_6_ and B_12_. Insufficient concentrations of these or an ineffective *MTHFR* enzyme can result in a decrease in methylation and in the higher production of toxic metabolites. For instance, vitamin B_12_ and folate are involved in the metabolic pathways of asymmetric dimethylarginine (ADMA) and symmetric dimethylarginine (SDMA), which are toxic amino acids that inhibit nitric oxide (NO) production. Thus, it can be hypothesized that folate and vitamin B_12_ deficiency results in high ADMA or SDMA levels and in the development of ADMA-related pathologies in individuals with obesity [22]. This can, at least in part, be explained by altered patterns of epigenetic modifications, together with the expansion of fat mass related to a greater concentration of serum lipid peroxides [37].

Variants of *MTHFR* can increase the genetic risk of obesity [38]. The functional integrity of the enzymes involved in folate/methionine cycles is necessary for the synthesis of the methyl-donor groups that are responsible for proper DNA methylation. DNA methylation is involved in regulating gene expression, including the leptin gene, which increases the risk of developing obesity [39]. However, the results of this study showed no statistical differences between obese and non-obese individuals in terms of the prevalence of the *MTHFR* C677T polymorphism, which is consistent with results obtained in a young population in Mexico [40], in 421 patients of Polish origin [41] and in a population of Brazilian patients [19]. The advantage of this study is that it involved patients with a wide range of BMIs, which allowed a comparison to be made of the distribution of genotypes of *MTHFR* C677T across four large groups categorized according to BMI (BMI < 30; BMI: 30–34.99; BMI: 35–39.99; and BMI: ≥40). Our results suggest that the 677 TT genotype is not a risk factor for a higher BMI. However, there are a number of studies that show contradictory results [18,42,43]. Further studies on a larger number of patients are still necessary to clarify the role of the *MTHFR* C677T genotype in the development of obesity.

This study had some limitations. We have not considered other possible genetic and metabolic factors which are known to affect the balance between the folate cycle and the methionine cycle. Homocysteine concentrations were not measured, data on which could provide additional insight into 5-MTHF synthesis or bioavailability.

In conclusion, the *MTHFR* 677 TT genotype was not found to have a significant influence on BMI or the obesity risk in codominant, dominant or recessive inheritance models. Patients with obesity and with the *MTHFR* TT genotype had significantly lower folate concentrations. A disturbed balance between folate and vitamin B_12_ concentrations after significant weight loss in individuals with the *MTHFR* T677T genotype is suggested by the data. Further large-scale studies are still needed to confirm our results.

## Figures and Tables

**Table 1:** Clinical and biochemical characteristics of the study subjects.

Characteristics	BMI < 30	BMI ≥ 30	*p*
Number of subjects	518	1194	-
Gender (male/female)	167/351	381/813	0.893
Age (years)	45.3 ± 15.0	44.6 ± 11.4	0.271
Height (cm)	167.9 ± 9.3	168.8 ± 9.0	0.086
Weight (kg)	68.9 ± 12.9	117.6 ± 24.1	<0.001
BMI (kg/m^2^)	24.3 ± 3.3	41.2 ± 7.2	<0.001
Glucose (mg/dL)	87.5 ± 19.5	105.8 ± 32.5	<0.001
Total cholesterol (mg/dL)	177.1 ± 37.2	188.2 ± 40.20	<0.001
HDL-cholesterol (mg/dL)	57.4 ± 14.9	45.0 ± 11.9	0.007
LDL-cholesterol (mg/dL)	107.6 ± 34.5	112.2 ± 35.2	<0.001
Triglycerides (mg/dL)	118.3 ± 136.5	157.6 ± 88.1	<0.001
Systolic blood pressure (mm Hg)	122.1 ± 15.6	134.9 ± 18.0	<0.001
Diastolic blood pressure (mm Hg)	76.4 ± 10.3	79.3 ± 10.6	<0.001

Data are presented as means ± standard deviations (SD); BMI: body mass index, HDL, high-density lipoprotein; LDL, low-density lipoprotein cholesterol. Chi-square, Student’s and Mann–Whitney tests were performed where appropriate.

**Table 2:** Distribution of BMI (body mass index) in studied adults, n = 1712, stratified by *MTHFR* C677T genotype.

Genotypes *MTHFR* C677T	Cohort n (%)	HWE	BMI (kg/m^2^) Mean ± SD	95% CI	Statistics *p*-Value *
	Total n = 1712				
TT	148 (8.6)		35.3 ± 9.8	33.7–36.9	
CT	752 (44.0)	0.159	35.6 ± 9.8	34.8–36.2	0.06
CC	812 (47.4)		36.7 ± 10.2	35.9–37.4	
	BMI < 30 n = 518				
TT	51 (10)		25.2 ± 3.50	24.3–26.2	
CT	235 (45)	0.531	24.1 ± 3.28	23.6–24.5	0.07
CC	232 (45)		24.4 ± 3.33	24.0–25.0	
	Class I obesity (BMI: 30–34.99) n = 257				
TT	27 (11.0)		32.6 ± 1.7	31.9–33.3	
CT	112 (44)	0.980	32.2 ± 1.8	32.3–32.8	0.59
CC	117 (46.0)		32.1 ± 1.4	32.1–32.6	
	Class II obesity (BMI: 35–39.99) n = 298				
TT	24 (8)		37.6 ± 1.5	36.9–38.2	
CT	137 (46)	0.202	37.5 ± 1.5	37.3–37.8	0.189
CC	137 (46)		37.2 ± 1.4	37.0–37.5	
	Class III obesity (BMI: ≥40) n = 640				
TT	46 (7)		46.9 ± 6.0	45.2–48.7	
CT	268 (42)	0.366	45.9 ± 5.0	45.3–46.5	0.162
CC	326 (51)		46.7 ± 5.9	46.1–47.4	

* ANOVA; BMI: body mass index; CI: confidence interval; HWE—Hardy–Weinberg equilibrium.

**Table 3:** Association of the *MTHFR* C677T polymorphism with obesity risk.

*MTHFR* C677T	BMI ≥ 30 (n = 1194) n (%)	BMI < 30 (n = 518) n (%)	Crude OR (95% CI)	*p*-Value	Adjusted OR ^#^ (95% CI)	*p*-Value
Codominant model						
CC	580 (49)	232 (45)	1.00	-	-	-
CT	517 (43)	235 (45)	0.88 [0.71–1.09]	0.248	0.88 [0.71–1.09]	0.251
TT	97 (8)	51 (10)	0.76 [0.52–1.03]	0.149	0.83 [0.55–1.26]	0.340
Dominant model						
CC	580	232	1.00	-	-	-
TT + CT vs. CC	614	286	0.86 [0.70–1.06]	0.149	0.94 [0.74–1.18]	0.579
Recessive model						
CC + CT	1097	467	1.00	-	-	
TT	97	51	0.81 [0.57–1.16]	0.249	0.86 [0.57–1.28]	0.453
Allelic model (T/C)	0.30/0.70	0.25/0.75	0.88 [0.75–1.03]	0.103	0.96 [0.75–1.23]	0.725

Abbreviations: BMI, body mass index; MTHFR, methylenetetrahydrofolate reductase; OR, odds ratio; CI, confidence intervals; ^#^ adjusted for potential confounders, including hypertension, diabetes dyslipidemia.

**Table 4:** The characteristics of patients with BMI ≥ 35 kg/m^2^ according to *MTHFR* C677T genotype and folate status.

	*MTHFR* C677T Polymorphism			
	TT n = 69	CT n = 392	CC n = 447	ANOVA/Kruskal–Wallis Tests *p*
Variables	Mean ± SD	Mean ± SD	Mean ± SD	
Age in years	43.7 ± 11.7	44.6 ± 11.7	43.8 ± 10.4	0.561
Weight (kg)	123.7 ± 24.4	122.0 ± 22.3	124.6 ± 23.6	0.276
BMI	43.3 ± 7.2	42.7 ± 6.2	43.6 ± 7.0	0.139
Fat %	43.7 ± 6.1	44.0 ± 6.6	44.2 ± 6.2	0.845
Fat mass (kg)	50.3 ± 12.3	52.0 ± 35.3	52.8 ± 25.7	0.821
Systolic blood pressure (mm Hg)	140.1 ± 22.8	135.7 ± 18.6	135.8 ± 18.3	0.236
Diastolic blood pressure (mm Hg)	79.5 ± 11.5	77.6 ± 10.1	77.4 ± 10.1	0.361
Fasting glucose (mg/dL)	108.1 ± 29.5	107.5 ± 33.5	108.4 ± 35.8	0.931
HbA1c (%)	6.1 ± 1.2	6.1 ± 1.3	6.2 ± 1.6	0.601
Total cholesterol (mg/dL)	183.6 ± 38.1	184.9 ± 39.8	188.8 ± 39.5	0.754
LDL-cholesterol (mg/dL)	107.3 ± 34.8	108.9 ± 35.4	110.8 ± 34.1	0.602
HDL-cholesterol (mg/dL)	45.1 ± 12.6	44.6 ± 12.3	44.1 ± 10.8	0.709
Triglycerides (mg/dL)	156.3 ± 72.8	156.9 ± 87.6	156.1 ± 75.3	0.988
Alanine aminotransferase (U/L)	45.13 ± 23.2	48.5 ± 28.4	47.5 ± 47.4	0.785
Aspartate aminotransferase (U/L)	27.5 ± 19.2	29.5 ± 18.7	28.5 ± 29.6	0.766
B_12_ (pg/mL)	320.6 ± 147.1	338.4 ± 149.3	329.9 ± 129.4	0.526
Folate (ng/mL)	6.6 ± 2.9	8.0 ± 3.7	8.2 ± 3.7	0.002
IL-6 (pg/mL)	3.1 ± 2.2	3.6 ± 5.1	3.3 ± 3.4	0.590
ESR	16.5 ± 12.1	16.7 ± 12.4	16.3 ± 13.4	0.919
CRP (g/L)	9.0 ± 8.1	9.4 ± 14.1	9.0 ± 7.9	0.875
CRE (mg/dL)	0.81 ±0.13	0.86 ±0.2	0.85 ±0.2	0.196
25(OH)D (ng/mL)	19.51 ± 7.9	19.3 ± 10.8	18.8 ± 9.7	0.750

The values are given as the means ± standard deviations (SD). Abbreviations: BMI, body mass index; HbA1c, glycosylated hemoglobin; LDL-cholesterol, low-density lipoprotein cholesterol; HDL-cholesterol, high-density lipoprotein cholesterol; ESR, erythrocyte sedimentation rate; CRP, C-reactive protein; CRE, creatinine. *p*-values were considered significant when *p* < 0.05.

**Table 5:** Changes in anthropometric and biochemical parameters following radical weight reduction according to *MTHFR* C677T genotype.

	*MTHFR* C677T Polymorphism			
Variables	TT n = 27	CT n = 154	CC n = 173	(ANOVA Tests) *p*
ΔBMI (kg/m^2^)	−12.9 ± 4.4	−12.8 ± 4.1	−13.8 ± 4.2	0.063
ΔBW (kg)	−36.5 ± 14.4	−36.4 ± 12.2	−39.2 ± 12.1	0.107
WL (%)	−30.7 ± 9.4	−30.5 ± 8.6	−31.6 ± 7.4	0.435
ExWL %	75.4 ± 23.8	75.2 ± 24.3	73.2 ± 22.3	0.708
Δ B_12_ (pg/mL)	−52.7 ± 165.6	−26.3 ± 186.9	−20.19 ± 132.1	0.617
Δ Folate (ng/mL)	3.03 ± 5.4	0.14 ± 5.18	0.66 ± 5.61	0.043

Δ, change (final–baseline); BW, body weight; WL %, weight loss percentage defined as 100 × (final-baseline)/baseline; ExWL %, excess weight loss percentage, defined as preoperative weight minus follow-up weight, divided by the excess weight and multiplied by 100; BMI, body mass index. Data expressed as mean ± SD.

## Data Availability

The data that support the findings of this study are available from the corresponding author upon reasonable request.
